# Clinical and Demographic Features of Primary Biliary Cholangitis in Kazakhstan

**DOI:** 10.3390/diseases14030112

**Published:** 2026-03-20

**Authors:** Aisulu Gainutdin, Alexander Nersesov, Komori Atsumasa, Aigul Raissova, Saltanat Madenova, Laura Yerdaliyeva, Dinara Suleimenova, Balday Issenova

**Affiliations:** 1 Asfendiyarov Kazakh National Medical University, Almaty 05000, Kazakhstan; alexander.nersesov@gmail.com (A.N.); raissova.23@gmail.com (A.R.); saltanatmadenova39@gmail.com (S.M.); lavrusha0708@gmail.com (L.Y.); s.dinara.s68@gmail.com (D.S.); 2 NHO Nagasaki Medical Center, Nagasaki 856-8562 , Japan; komoripowers@me.com

**Keywords:** primary biliary cholangitis, autoimmune hepatitis, epidemiology, Kazakhstan

## Abstract

Background/Objectives: Primary biliary cholangitis (PBC) is a chronic immune-mediated cholestatic liver disease with increasing global prevalence. However, data on this disease from Central Asia are lacking. We aimed to describe the clinical, serological, and treatment characteristics of PBC patients in Kazakhstan. Methods: This study was a multicenter, retrospective, observational study including adults diagnosed with PBC between 2014 and 2022 across seven hepatology centers in Kazakhstan. Clinical presentation, laboratory parameters, autoimmune comorbidities, liver disease severity, and ursodeoxycholic acid (UDCA) treatment response were assessed. Biochemical response at 1 year was evaluated using Paris-1 and Barcelona criteria. Results: A total of 230 patients were included; 93.9% were female and 91.3% were of Asian ethnicity, with a median age at diagnosis of 53 years. Cirrhosis was present at diagnosis in 50.2% of the patients. PBC with autoimmune hepatitis (AIH) features was identified in 56.1% of the patients and was associated with higher rates of cirrhosis, portal hypertension complications, antinuclear antibody (ANA) positivity, and higher elastography indices compared with isolated PBC. Overall, approximately 55% of the patients achieved a biochemical response to UDCA at 1 year, with similar response rates between patients with PBC and those with PBC with AIH features. Conclusions: This first comprehensive study of PBC in Kazakhstan demonstrates late disease presentation with a high burden of cirrhosis and frequent AIH features. Despite advanced disease, about half of the patients achieved biochemical remission on UDCA. These findings underscore the need for earlier diagnosis and optimized management strategies for PBC in Kazakhstan and similar settings in Central Asia.

## 1. Introduction

Primary biliary cholangitis (PBC) is a chronic immune-mediated cholestatic liver disease with rising incidence and prevalence worldwide [1]. Recent global meta-analyses estimate a pooled prevalence of approximately 18 per 100,000 persons and an incidence of 1.8 per 100,000 person-years, with considerable variability across regions [2]. The rates are lowest in the Asia–Pacific region and higher in Europe and North America [3,4]. PBC also commonly overlaps with other autoimmune diseases: up to 73% of patients have extrahepatic autoimmune manifestations—most often Sjögren’s syndrome, autoimmune thyroid disease, or systemic sclerosis [5].

In contemporary national cohorts from the Netherlands and Sweden, PBC shows increasing prevalence over time and female predominance; it is typically diagnosed in the fifth to seventh decades of life, often with early-stage biochemical abnormalities [6,7]. In recent years, improved diagnostics have revealed rising PBC incidence in Asian populations. Despite these insights, epidemiological and clinical data from Central Asia remain nonexistent.

Kazakhstan, a large upper-middle-income country in Central Asia with changing health-system capacities, has no published descriptive studies of PBC. Given ethnic, environmental, and healthcare-access differences, data from Europe and North America may not reflect the clinical profile of PBC in Kazakhstan. Understanding local patterns of disease presentation, biochemical severity, autoimmune comorbidities, and treatment response is critical for establishing evidence-based management pathways. To date, published data on primary biliary cholangitis in Kazakhstan are limited to a small interventional cohort of advanced ursodeoxycholic acid (UDCA)-refractory patients and isolated case reports, with no comprehensive descriptive studies reporting demographic, immunologic, and clinical characteristics of PBC patients in routine hepatology practice [8,9,10,11,12].

Therefore, we conducted a descriptive study of patients with PBC across multiple centers in Kazakhstan. The primary objective of this study was to describe the profile of Kazakh PBC patients, including disease presentation, comorbidities, and treatment outcomes. In addition, we hypothesized that due to healthcare and demographic differences, many patients are diagnosed at later disease stages than European and North American cases. Thus, the secondary objective was to compare patients with isolated PBC to those with PBC with autoimmune hepatitis (AIH) features to elucidate any differences in presentation or response to therapy.

## 2. Materials and Methods

### 2.1. The Study Population

We conducted a multicenter retrospective observational study of adult patients (≥18 years) with confirmed PBC across seven hepatology centers in Kazakhstan. The study period was January 2014 through May 2022.

A diagnosis of PBC was considered if the patient fulfilled at least two of the following diagnostic criteria, as recommended by the American Association for the Study of Liver Disease guidelines: (1) positive antimitochondrial antibody (AMA) serology (titer ≥ 1:40 by immunofluorescence); (2) a persistent increase in serum alkaline phosphatase (ALP) levels; and (3) liver histology showing nonsuppurative lymphocytic cholangitis with interlobular bile duct damage [13]. The diagnosis was established based on serological data. All patients were examined by ultrasound and indirect liver elastography. PBC with AIH features was established using ANA positivity (ANA ≥ 1:160) in the diagnostic titer in patients with PBC.

Patients in whom the diagnosis could not be confirmed or who had another etiology of liver disease were excluded. AMA status was assessed by indirect immunofluorescence. All AMA-positive patients had titers ≥ 1:40. All centers adhered to uniform diagnostic criteria and data collection protocols.

### 2.2. Data Collection

De-identified clinical data were extracted retrospectively from medical records at each center by trained physicians. Collected variables included demographics (age, sex, ethnicity), lifestyle factors (smoking, alcohol use), diagnosis details (year of first symptoms, year of PBC diagnosis, and presence of AIH features), and relevant comorbidities (other autoimmune disorders such as Hashimoto thyroiditis, Sjögren’s syndrome, rheumatoid arthritis, or CREST syndrome). Baseline liver disease status (cirrhosis and any features of portal hypertension) at diagnosis was recorded, as were key symptoms (pruritus, fatigue, or peripheral edema). Liver cirrhosis was diagnosed based on laboratory and instrumental examinations in accordance with standard diagnostic guidelines. Laboratory data included liver enzymes—alanine transferase (ALT), aspartate aminotransferase (AST), alkaline phosphatase (ALP), and gamma-glutamyltransferase (GGT)—as well as total bilirubin and immunoglobulins, when available. Serological AMA (including M2 subtype) and antinuclear antibody (ANA) status was recorded for each patient. The presence and titer of AMAs were assessed by indirect immunofluorescence (titer ≥ 1:40 considered positive, in line with diagnostic standards). Data on PBC treatment with UDCA, including the weight-based dose, were collected. All patients were managed with standard-dose UDCA (target dose 13–15 mg/kg/day) as first-line therapy. Treatment response was assessed using the efficacy criteria recommended by the European guidelines for the management of primary biliary cholangitis. Treatment adherence was noted (fully adherent vs. dose interruptions). For patients on UDCA for ≥12 months, we assessed the biochemical response to therapy at year one. Biochemical response was evaluated by two widely used criteria: the Paris-1 criterion and the Barcelona criterion. Patients meeting either criterion at 1 year were classified as responders under that definition. The duration of follow-up was defined from the date of diagnosis to the last recorded clinic visit for each patient.

### 2.3. Ethics

All procedures were conducted in accordance with the ethical standards of the Helsinki Declaration, and the study was approved by the local ethical committee of Asfendiyarov Kazakh National Medical University (protocol number 3(94), approval date 25 March 2020).

### 2.4. Statistical Analysis

Continuous variables were assessed for normality (Shapiro–Wilk test). Normally distributed continuous variables are presented as mean ± standard deviation (SD), and non-normal variables as median with interquartile range (IQR). Categorical variables are summarized as counts and percentages. Group comparisons of patients with PBC alone vs. those with PBC with AIH features were conducted using the chi-square test or Fisher’s exact test for categorical variables, and Student’s *t*-test or Wilcoxon two-sample test for continuous variables, as appropriate. Pairwise deletion was applied to missing data. A two-tailed *p* < 0.05 was considered statistically significant. All statistical analyses were performed using SAS OnDemand for Academics (version 3.81, Cary, NC, USA).

## 3. Results

As shown in Table 1, a total of 230 patients were included in the analysis. The cohort was predominantly Asian (91.3%) and female (93.9%). The prevalence of current or past smoking and alcohol consumption was low (4.6% and 2.3%, respectively). Regarding diagnosis, 56.1% of the patients had PBC with AIH features, while 43.9% had PBC alone. Cirrhosis was present in approximately half of the cohort (50.2%). Features of portal hypertension were common, with ascites observed in 16.2%, esophageal varices in 29.8%, and hepatic encephalopathy in 24.5% of the patients. Extrahepatic autoimmune conditions were observed in a subset of the patients, including Hashimoto’s thyroiditis (13.0%), Sjögren’s syndrome (8.7%), rheumatoid arthritis (4.8%), and CREST syndrome/scleroderma (2.6%). Disease-related symptoms were frequent: pruritus was observed in 64.8% of the patients and fatigue in 79.0%, while peripheral edema was present in 16.4%. Serologically, AMA or AMA-M2 positivity was detected in 78.4% of the patients, and ANA positivity in 43.6%. Most patients demonstrated good treatment adherence, with 82.4% fully adhering to therapy. The median age of the cohort was 55.5 years, with a median age at diagnosis of 53 years. The median body mass index was 23.81 kg/m^2^, and the median time to diagnosis was 16 months. Disease severity indices were generally low to moderate, with a median elastography index of 3. Liver biochemistry indices were highly variable, with median ALP, ALT, and GGT indices of 270, 51.7, and 151.1, respectively. The mean daily dose of ursodeoxycholic acid was 1000 mg, while non-invasive fibrosis scores showed substantial dispersion (FIB-4: 2.03; APRI: 0.80).

According to Table 2, baseline demographic characteristics were comparable between patients with PBC and those with PBC with AIH features. Cirrhosis was more frequent in the PBC-with-AIH-features group than in the PBC-alone group (59.7% vs. 37.4%, *p* = 0.0012). Similarly, hepatic encephalopathy (30.2%, *p* = 0.0209) and esophageal varices (37.2%, *p* = 0.0054) were more common in patients with PBC with AIH features than in those with PBC alone, whereas the prevalence of ascites did not differ between groups (*p* = 0.9342). The frequency of extrahepatic autoimmune diseases, including Hashimoto’s thyroiditis, Sjögren’s syndrome, rheumatoid arthritis, and CREST syndrome, was comparable between groups (all *p* > 0.05). Pruritus, fatigue, and peripheral edema were common in both groups, with no statistically significant differences. Serological profiles showed a marked contrast: the prevalence of ANA positivity was substantially higher in patients with PBC with AIH features (63.0% vs. 15.7%, *p* < 0.0001), while AMA/AMA-M2 positivity did not differ significantly between the groups. Treatment adherence was high and comparable. Patients with PBC with AIH features demonstrated higher indices of liver disease severity, with a median elastography index of 4 vs. 2 (*p* = 0.0004). Among biochemical parameters, AST levels were modestly higher in patients with PBC with AIH features (*p* = 0.0451), while ALT, ALP, GGT, and ursodeoxycholic acid dose were similar between the two groups. Non-invasive fibrosis scores showed a trend toward higher values in patients with PBC with AIH features, with APRI approaching statistical significance (*p* = 0.0542).

As shown in Table 3, at 1-year of follow-up, overall biochemical response rates were consistent between Paris-1 and Barcelona (55.77% vs. 55.09%, respectively). Similarly, at 1 year, biochemical response rates were comparable between patients with PBC and those with PBC with AIH features using both Paris-1 (57.3% vs. 54.6%) and Barcelona criteria (53.9% vs. 56.0%).

## 4. Discussion

This study provides the first comprehensive analysis of PBC patients in Kazakhstan, revealing both expected consistencies with and notable differences from international data. We found that Kazakh PBC patients are predominantly female with a mean diagnosis age in the early 50s, which is consistent with the known epidemiology of PBC [14]. The majority of our patients had no history of alcohol overuse, and few were smokers, aligning with reports that PBC etiology is unrelated to these traditional liver risk factors and may instead be linked to other environmental triggers, such as prior infections, in genetically susceptible individuals [15,16].

Despite these similarities, our findings also highlight important differences in disease presentation that likely reflect healthcare and genetic factors unique to this region. Notably, half of our cohort had cirrhosis at the time of PBC diagnosis. This rate is substantially higher than that reported in contemporary studies from North America or Europe, where only about 10–20% of patients had cirrhosis at diagnosis due to earlier detection of asymptomatic cases [17]. The high prevalence of advanced fibrosis in our patients suggests that PBC is often recognized late in its course in Kazakhstan. Possible contributing factors include limited routine liver enzyme screening, delayed referral of asymptomatic cholestatic lab abnormalities, or reduced awareness, leading patients to present only once symptoms (fatigue, pruritus) or complications (ascites, variceal bleeding) develop.

Another striking finding is the high proportion of PBC with AIH features in our cohort. Features were identified in 56% of patients, far exceeding the 5–15% frequency reported in most Western series [18,19,20]. This discrepancy may have several explanations. First, the advanced stage of disease at presentation could predispose patients to PBC with AIH features. There is evidence that PBC patients can develop features of AIH over time if cholestatic injury is longstanding [21,22]. In our cohort, the long diagnostic delay and high rate of cirrhosis mean that many patients had prolonged uncontrolled cholestasis, which may have triggered or unmasked autoimmune hepatitis features by the time they were diagnosed. From a practical standpoint, the high frequency of patients presenting with PBC with AIH features in Kazakhstan calls for vigilance in monitoring PBC patients for hepatic activity.

The patients with PBC with AIH features in our study had a significantly higher ANA positivity rate and more aggressive disease than patients with isolated PBC. This aligns with reports that patients with PBC with AIH features often experience a more severe course than those with PBC or AIH alone [23]. Nonetheless, when treated appropriately, their outcomes can be favorable. In our cohort, patients with PBC with AIH features did not show a higher 1-year treatment failure rate compared to those with PBC alone, suggesting that UDCA therapy was beneficial.

Our study also sheds light on the serological profile of PBC in our population. We observed a somewhat lower prevalence of AMA positivity (78% overall) than is typically described in European/North American cohorts, where 90–95% of PBC patients are AMA-positive [24]. Notably, AMA-negative PBC has been reported at higher rates in some East Asian studies, up to 15% in China [25], and our finding of 22% AMA negativity is within the context of a predominantly Asian (Kazakh) cohort.

The first-year treatment response to UDCA in our cohort was modest and aligns with international experience. By either Paris-1 or Barcelona criteria, about 55% of patients achieved a complete biochemical response after 1 year on UDCA. This implies that 45% of patients had an inadequate response, a proportion in line with the upper range of figures reported globally; in Western cohorts, 20–40% are UDCA non-responders, depending on the criteria [26,27]. The lack of a difference in UDCA response rates between patients with PBC with AIH features and those with pure PBC in our study is notable. This could be partly because patients with PBC with AIH features who respond to UDCA represent those with less aggressive bile duct disease, hence surviving long enough to manifest AIH features.

The results offer a benchmark for clinicians in Central Asia regarding what patient profiles to expect; the findings also highlight areas for improvement in patient monitoring. We also acknowledge some limitations: the retrospective design may introduce information bias (e.g., under-reporting of mild symptoms or comorbidities), and missing data for certain variables (IgG levels) required us to rely on available subsets for analysis. Furthermore, while we compared PBC with and without AIH features, we did not have a separate control group with isolated AIH—thus, our conclusions on AIH features are specific to differences from classic PBC, not from pure AIH. Lastly, genetic studies were beyond our scope, but future research might investigate whether unique genetic or environmental factors underlie the high rate of PBC and AIH features observed.

In a broader context, our findings reinforce that PBC in Kazakhstan shares many features with PBC elsewhere, but patients here are diagnosed later in the disease course. This has clinical implications: there is a window of opportunity to intervene earlier, which could be achieved by raising awareness among primary care and general physicians to check cholestatic liver enzymes and AMAs in patients (particularly middle-aged women) with vague symptoms, like fatigue or pruritus. The association of PBC with other autoimmune diseases also means that multidisciplinary collaboration (with endocrinologists, rheumatologists, etc.) is important for comprehensive care.

## 5. Conclusions

In conclusion, this study is the first to characterize the clinical profile of primary biliary cholangitis in Kazakhstan, revealing a disease that predominantly affects middle-aged women, often presents at an advanced stage, and frequently occurs with AIH features. Our PBC cohort had a high rate of cirrhosis and autoimmune features at diagnosis, likely reflecting delayed case detection and perhaps unique regional factors. Nevertheless, about half of the patients achieved biochemical remission on UDCA, consistent with global experience, while the remainder required additional therapies to improve their prognosis. These findings highlight an urgent need for earlier diagnosis and intervention in PBC patients in Kazakhstan and similar settings in Central Asia. By increasing awareness of PBC among healthcare providers and ensuring availability of second-line treatments for UDCA non-responders, we can potentially change the disease course—improving survival and quality of life for PBC patients in this region. Ultimately, our study underscores that while PBC is a global disease with well-established therapies, local population characteristics and healthcare infrastructure greatly influence patient outcomes, and strategies tailored to the regional context are essential for optimizing care.

## Figures and Tables

**Table 1:** Baseline characteristics of patients (*n* = 230).

Characteristics	Frequency	Percent (%)
Ethnicity		
Asian	210	91.30
Other	20	8.70
Gender		
Female	216	93.91
Male	14	6.09
Main diagnosis		
PBC	101	43.91
PBC with AIH features	129	56.09
Smokers	10	4.59
Alcohol consumers	5	2.29
Cirrhosis, yes	108	50.23
Ascites, yes	37	16.23
Hepatic encephalopathy, yes	56	24.45
Esophageal varices, yes	68	29.82
Hashimoto’s thyroiditis, yes	30	13.04
Sjögren’s syndrome, yes	20	8.70
Rheumatoid arthritis, yes	11	4.78
CREST syndrome/scleroderma, yes	6	2.61
Pruritus, yes	147	64.76
Fatigue, yes	180	78.95
Peripheral edema, yes	37	16.37
AMA/AMA-M2		
Negative	46	21.60
Positive	167	78.40
ANA		
Negative	114	56.44
Positive	88	43.56
Treatment adherence		
Adhered fully	168	82.35
Adhered with violations	36	17.65
Age, years	55.5 (18–84)	17 *
Age at diagnosis, years	53 (18–84)	15 *
BMI, kg/m^2^	23.82 (16.4–46)	4.65 *
Time to diagnosis, months	16 (0–132)	24 *
Elastography, kPa	3 (0–4)	3 *
ALT, U/L	51.74 (8.09–641)	59.9 *
AST, U/L	55.9 (9.7–288)	50.5 *
ALP, U/L	270 (45–1815)	296.6 *
GGT, U/L	151.1 (10–1521)	208 *
Ursodeoxycholic acid, mg	1000 (500–2250)	250 *
FIB 4	2.03 (0.37–26.19)	2.46 *
De Ritis	1 (0.36–16.33)	0.51 *
APRI	0.80 (0.10–13.62)	0.98 *

Values are presented as no. (%) and median (min–max); PBC, primary biliary cholangitis; PBC + AIH, primary biliary cholangitis with autoimmune hepatitis features; AMA/AMA-M2, antimitochondrial M2 antibody; ANA, antinuclear antibody; BMI, body mass index; ALT, alanine transaminase; AST, aspartate aminotransferase; ALP, alkaline phosphatase; GGT, gamma-glutamyl transferase; SD, standard deviation; *, IQR (interquartile range).

**Table 2:** Characteristics of patients with primary biliary cholangitis and those with primary biliary cholangitis with autoimmune hepatitis features (*n* = 230).

Characteristics	PBC	PBC+ AIH Features	*p*-Value
Ethnicity			0.9184
Asian	92 (91.09)	118 (91.47)	
Other	9 (8.91)	11 (8.53)	
Gender			0.9345
female	95 (94.06)	121 (93.80)	
male	6 (5.94)	8 (6.20)	
Smoking			0.2550
no	87 (95.60)	121 (95.28)	
yes	4 (4.40)	6 (4.72)	
Alcohol consumption			0.2350
no	91 (98.91)	122 (96.83)	
yes	1 (1.09)	4 (3.17)	
Cirrhosis			0.0012
no	57 (62.64)	50 (40.32)	
yes	34 (37.36)	74 (59.68)	
Ascites			0.9342
no	84 (84.00)	107 (83.59)	
yes	16 (16.00)	21 (16.41)	
Hepatic encephalopathy			0.0209
no	83 (83.00)	90 (69.77)	
yes	17 (17.00)	39 (30.23)	
Esophageal varices			0.0054
no	79 (79.80)	81 (62.79)	
yes	20 (20.20)	48 (37.21)	
Hashimoto’s thyroiditis			0.7445
no	87 (86.14)	113 (87.60)	
yes	14 (13.86)	16 (12.40)	
Sjögren’s syndrome			0.4006
no	94 (93.07)	116 (89.92)	
yes	7 (6.93)	13 (10.08)	
Rheumatoid arthritis			0.2406
no	96 (95.05)	123 (95.35)	
yes	5 (4.95)	6 (4.65)	
Crest syndrome/scleroderma			0.2888
no	99 (98.02)	125 (96.90)	
yes	2 (1.98)	4 (3.10)	
Pruritus			0.4766
no	32 (32.65)	48 (37.21)	
yes	66 (67.35)	81 (62.79)	
Fatigue			0.0909
no	26 (26.26)	22 (17.05)	
yes	73 (73.74)	107 (82.95)	
Peripheral edema			0.1587
no	85 (87.63)	104 (80.62)	
yes	12 (12.37)	25 (19.38)	
AMA/AMA-M2			0.1434
negative	24 (26.37)	22 (18.03)	
positive	67 (73.63)	100 (81.97)	
ANA			<0.0001
negative	70 (84.34)	44 (36.97)	
positive	13 (15.66)	75 (63.03)	
Treatment adherence			0.9827
adhered fully	75 (82.42)	93 (82.30)	
adhered with violations	16 (17.58)	20 (17.70)	
Age, years	55 (20–75)	56 (18–84)	0.4091
Age at diagnosis, years	53.5 (20–75)	53 (18–84)	0.6808
BMI, kg/m^2^	23.70 (16.44–34.14)	23.95 (16.49–46.00)	0.6579
Time to diagnosis, months	16 (0–132)	16 (0–132)	0.7074
Elastography, kPa	2 (0–4)	4 (0–4)	0.0004
ALT, U/L	46.99 (8.09–418)	52.8 (9–641)	0.1206
AST, U/L	47.22 (9.7–288)	61.72 (15.2–282)	0.0451
ALP, U/L	265.55 (45–955)	275 (65–1815)	0.6764
GGT, U/L	164.7 (12.56–1086)	137 (10–1521)	0.896
Ursodeoxycholic acid dose, mg	1000 (500–2250)	1000 (500–1500)	0.7931
FIB-4	1.80 (0.38–17.68)	2.14 (0.37–26.19)	0.1522
DE RITIS	0.99 (0.36–16.33)	1.03 (0.39–3.83)	0.5575
APRI	0.64 (0.10–7.98)	0.88 (0.14–13.63)	0.0542

Values are presented as no. (%) and median (min-max); PBC, primary biliary cholangitis; PBC + AIH, primary biliary cholangitis with autoimmune hepatitis features; AMA/AMA-M2, antimitochondrial M2 antibody; ANA, antinuclear antibody; BMI, body mass index; ALT, alanine transaminase; AST, aspartate aminotransferase; ALP, alkaline phosphatase; GGT, gamma-glutamyl transferase; SD, standard deviation.

**Table 3:** Overall and group responses to ursodeoxycholic acid treatment at 1 year according to Paris 1 and Barcelona criteria.

Response Criteria	Overall Response at 1 Year		Response at 1 Year			
			PBC		PBC with AIH Features	
	Available data (*n*)	Cohort Response *n* (%)	Available data (*n*)	Cohort Response *n* (%)	Available data (*n*)	Cohort Response *n* (%)
Paris-1	208	116 (55.77)	89	51 (57.30)	119	65 (54.62)
Barcelona	216	119 (55.09)	91	49 (53.85)	125	70 (56.00)

Values are presented as no. (%); PBC, primary biliary cholangitis; AIH, autoimmune hepatitis.

## Data Availability

The data presented in this study are available on request from the corresponding author due to institutional and national data protection regulations that restrict public sharing of health data.

## Abbreviations

The following abbreviations are used in this manuscript:

PBC	Primary biliary cholangitis
PBC + AIH	Primary biliary cholangitis with autoimmune hepatitis features
UDCA	Ursodeoxycholic acid
AIH	Autoimmune hepatitis
AST	Aspartate aminotransferase
ALT	Alanine transaminase
ALP	Alkaline phosphatase
GGT	Gamma-glutamyl transferase
IQR	Interquartile range
SD	Standard deviation
AMA/AMA-M2	Antimitochondrial M2 antibody
ANA	Antinuclear antibody
BMI	Body mass index
